# “Science for All”: A Case Study of Digitizing Inquiry-Driven Professional Development

**DOI:** 10.4236/ce.2021.128134

**Published:** 2021-08-02

**Authors:** Timothy C. Indahl, Michael P. Ekker, Gregory M. Sindberg, Chris Pierret

**Affiliations:** 1 Center for Clinical and Translational Science, Mayo Clinic, Rochester, USA; 2 Integrated Science Education Outreach (InSciEd Out), Rochester, USA; 3 School of Education, Saint Mary’s University of Minnesota, Winona, USA; 4 Department of Biochemistry and Molecular Biology, Mayo Clinic, Rochester, USA

**Keywords:** Professional Development, Digitization, Inquiry, Technology

## Abstract

To adapt to the increasingly technology-driven environment of modern K-12 education Integrated Science Education Outreach (InSciEd Out) digitized an extensive professional development curriculum library that forms the core experience for teachers joining the program. In previous years the curriculum had been delivered solely in print form. The goals of this conversion were to better employ technology in the teacher training experience that mirrored best practice in their K-12 classrooms and to provide a more scalable product for InSciEd Out. The digitized professional development curriculum was delivered using Google Classroom accessed by teachers with Chromebooks. The digitization measurably improved flexibility for engagement in scientific experimentation and granted immediate access to course feedback for the program. Teachers who participated rated the course positively in general and specifically reported increased self-efficacy in technology use both in the internship and in the classroom.

## 1. Introduction

Science education lags behind scientific research practice in the utilization of technology, which limits opportunities for students to become producers of novel science (Sabelli, 2006). Inquiry has been shown to improve student learning outcomes in science compared with other methods (National Research Council, 2007). As a program, Integrated Science Education Outreach (InSciEd Out) creates partnerships between scientists with advanced technological laboratory tools and classroom teachers to improve the learning of science for all students (Pierret et al., 2012; Yang, LaBounty, Ekker, & Pierret, 2016). However, the professional development course for K-12 that InSciEd Out employs to train partner teachers has remained grounded in pencil and paper delivery.

Digitizing this professional development experience was a necessary step to enable technology use during the training experience and in the teachers’ eventual classroom implementation. Goals for this digitization effort included satisfaction with—and efficacy in using—technology within the professional development experience and in their own classrooms. The utilization of technology in classroom inquiry is intended to be an enhancement of student learning through automating less necessary procedures within student tasks (Quintana et al., 2004; Reiser, 2004). Success of technology in the classroom requires the teacher to be the driver of and model for its use (Miranda & Russell, 2012). Teachers often lack both confidence and practical experience in the use of technology for teaching and learning. In fact, teachers’ self-efficacy regarding technology integration in their classroom has been described as “inadequate” (Moore-Hayes, 2011). Further, pre-service and beginning teachers generally self-reported high efficacy in all areas, including classroom management and inclusion, with the specific exception of technology integration (Moore-Hayes, 2008).

Introducing current technology to teachers in the classroom can overcome this challenge (Cavanaugh & Dawson, 2010; Gerard, Varma, Corliss, & Linn, 2011; Moore-Hayes, 2011; Rehmat & Bailey, 2014). Specifically, a review of professional development studies showed that technology-enhanced professional development focused on inquiry significantly enhanced student outcomes when the initiative was sustained for over a year (Gerard et al., 2011).

Production of science through inquiry is a tenet of the InSciEd Out program, which has led to a sustained increase of student outcomes in the classroom (Pierret et al., 2012; Yang et al., 2016). Technology that has become available to classrooms through this partnership includes WiFi microscopes, advanced image capture and analysis, and cooperative working environments like the Google Suite.

The core professional development offering of InSciEd Out is a 12-day immersive course designed to engage teachers in inquiry (Pierret et al., 2012; Yang et al., 2016). The contents of the program’s professional development curriculum had been updated regularly, but the curriculum materials (including all lesson plans, worksheets, and assessments) had been printed and organized in binders to distribute to teacher learners and teaching facilitators (Table 1). This approach was cumbersome when inquiry-driven components required scheduling changes after the printing of materials.

Ultimately, it was deemed necessary to fully digitize the curriculum (including the participants interface with the materials). Revised learning goals for the teacher experience prioritized maximizing the technological skills that teachers were to gain and enable participants to more effectively utilize the science-based technologies in their own classrooms.

### Rationale

The following is a case study of this digitization process, including integration of learning technologies with research tools, such as microscopes, to enable teachers to capture experimental data on live research animals as part of their professional growth. We applied a quasi-experimental approach to: 1) help direct this digital revision of our core product; and 2) model teacher participants to access similar data in their own classrooms. We report it here for those organizations similar to InSciEd Out looking to transition their delivery strategy. Metrics of success for this case study included satisfaction with the technology used in the course and its impact on their intentions toward use in their own classrooms.

## 2. Methods

### 2.1. Professional Development Course

InSciEd Out held a professional development course in 2016 with 12-day of face-to-face learning similar to published methods (Hammerlund, Hoody, & Pierret, 2012; Pierret et al., 2012), which included inquiry-driven components such as working with Zebrafish and designing experiments. The 2016 course included 23 teachers from multiple schools in a Florida school district new to InSciEd Out programming. Digital delivery of the same curriculum was performed in the summer of 2017 with groups located in Minnesota, Florida and Illinois. Those courses included a total of 55 teachers from multiple schools in each location.

**Figure 1.**
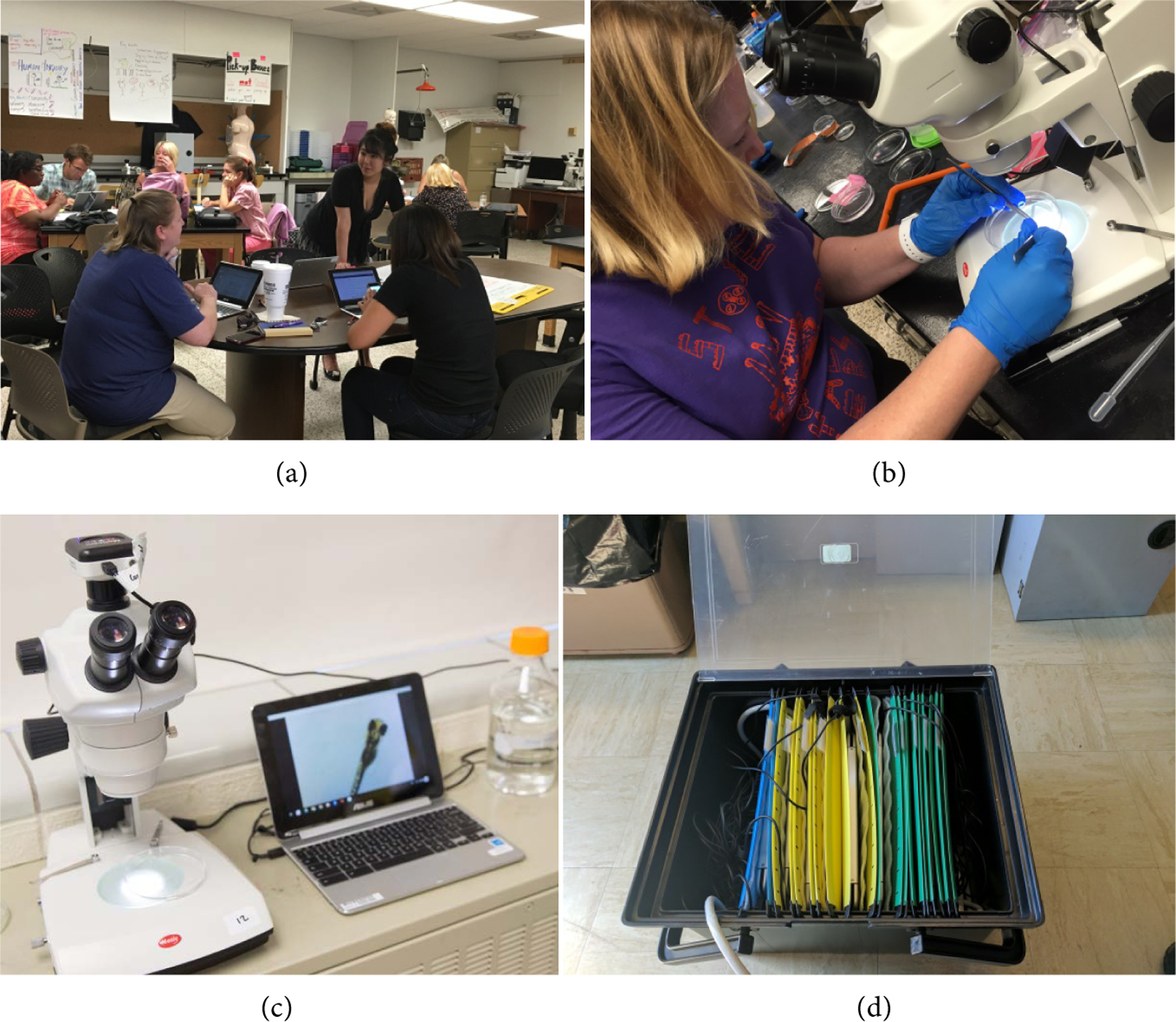
Photos of technology utilized during professional development. (a) Teachers utilizing Chromebooks in classrooms; (b) Teachers utilizing Motic microscopes equipped with WiFi cameras; (c) Motic microscope equipped with WiFi Camera and connected Chromebook; (d) Chromebook storage box.

All materials of the 2017 courses were presented digitally, including lesson plan access, data collection throughout the inquiry process, data analysis, assessments, and creation of scientific posters that, highlighted the teacher’s ability to practice their voices as scientists (Figure 1(a)).

### 2.2. Chromebooks

To standardize the experience and minimize troubleshooting steps, each teacher was given the use of an Asus C100P Chromebook for the duration of the professional development program. Advantages of these devices include long battery life (more than 8 hours), relatively low cost (under $200 at the time of purchase), a wide range of capabilities, and touchscreen and tablet functionality while also maintaining a usable keyboard for typing. In addition, their ready connection to the Google Classroom class management system and the microscopes used in the program made them attractive for use in this situation.

Sets of Chromebooks were stored in portable plastic file boxes containing hanging folders to corral each device within individually labeled sleeves (Figure 1(c)). Each box fit 10 Chromebooks and two 6-outlet power strips to support all devices’ charging needs on a single outlet, which is essential in most classroom environments. In addition, each box contained a flash drive with ChromeOS recovery media for addressing technological issues on site if they arose. While each box is relatively heavy fully loaded (about 20 lbs.), they can be easily transported in cars and on rolling carts of the type regularly available at schools.

### 2.3. Microscopes

Data was collected from experiments electronically using a variety of methods. One method was the use of dissecting microscopes paired with Moticam X WiFi cameras (Figure 1(b)). These cameras transmit live images from the microscope to up to five wireless devices by broadcasting its own wifi network. The Chromebooks noted in the previous section were connected to the cameras with the use of the MotiConnect application published on the Google Play Store to view the live feed. This application includes many features that were valuable for collecting experimental data. The features include picture and video recording, line and shape drawing on the images, as well as length, angle, and area measurement. The latter are critical measurements when assessing the impact of experimental variables on Zebrafish development.

### 2.4. E-Learning Platform

The Chromebooks’ integration with Google Apps, Google Classroom allowed an efficient and effective option to deliver the curriculum digitally. The InSciEd Out curriculum was converted to Google Classroom by creating a template class for each of the five topics, or threads, of the course, all of which were previously present in the paper binders: Genetics, Nature of Science, Pedagogy, Dialogue, and the health topic of focus for the given course (mental health for the 2017 courses). Each lesson and assignment was created within a master template (independent Google Classroom) for each thread, and then materials from all threads were copied into the Google Classroom for the course. The Google Classroom environment allowed for more fluid curriculum delivery that was responsive to the level and pace of the group, including customization of lessons and handouts, when needed. The template structure allowed this customization without making changes to the base curriculum, because it was safely housed in the separate Google Classroom template. Scheduling flexibility provided an authentic science experience, with schedule changes for the didactic curriculum able to be made as experiments progressed.

### 2.5. Assessment

Utilizing Chromebooks also enabled digitization of assessments, primarily through the use of Google Forms, but also through apps designed to allow drawing on PDF files.

Participant “talking drawings”, proved more challenging to digitize. The process of this assessment requires illustrating one’s viewpoint to an open-ended prompt through a drawing accompanied by written and/or spoken words (Chambers, 1983; Driessnack & Gallo, 2013; Koep et al., 2016). Because Google Drawings is not an intuitive platform for free drawing, Clarisketch app was selected. Clarisketch allows for freehand drawing, but was a challenge for the inclusion of text and voice, creating a need for a tutored walkthrough with course instructors. Clarisketch was not very effective from the point of view of the teacher participants, leading to a change to Autodesk SketchBook in 2017.

The new availability of Android apps on the Google Play Store in 2017 allowed a wider range of apps for the talking drawings. Autodesk SketchBook offers similar freehand drawing features, but was much more intuitive to the teachers to use. In addition, there are much simpler and more effective options for importing and exporting images to and from the app, which was one of the major challenges with Clarisketch. Autodesk SketchBook still required creation of protocol for teachers to learn how to use it effectively for talking drawings, but overall it seemed much more intuitive and effective than Clarisketch.

## 3. Results

The 2017 12-day course was delivered at three sites across the United States, and included 55 teachers from across those sites. The course ran smoothly throughout the 12 days, with all teachers utilizing Chromebooks despite a few of the teachers being outspoken toward their concern of utilizing technology, specifically that it was more complex and uncomfortable. On the final day of the 12-day professional development, teachers were asked to provide feedback on the course in two assessments: a course evaluation and a technology satisfaction survey. These surveys were collected anonymously via Google Forms.

### 3.1. Course Evaluations: Course Ratings

The course evaluation was administered to gauge overall effectiveness of the course, and included 5 items about various aspects of the course itself (Table 2). 55 teachers that attended the course completed this assessment, and were asked to indicate their satisfaction on a 5-point Likert-type scale ranging from “Strongly Disagree” (1) to “Strongly Agree” (5). Teachers were also asked to give an overall rating of the course, from “Very Bad” (1) to “Excellent” (5). Mean rating for the course was 4.51.

### 3.2. Technology Satisfaction

**Figure 2.**
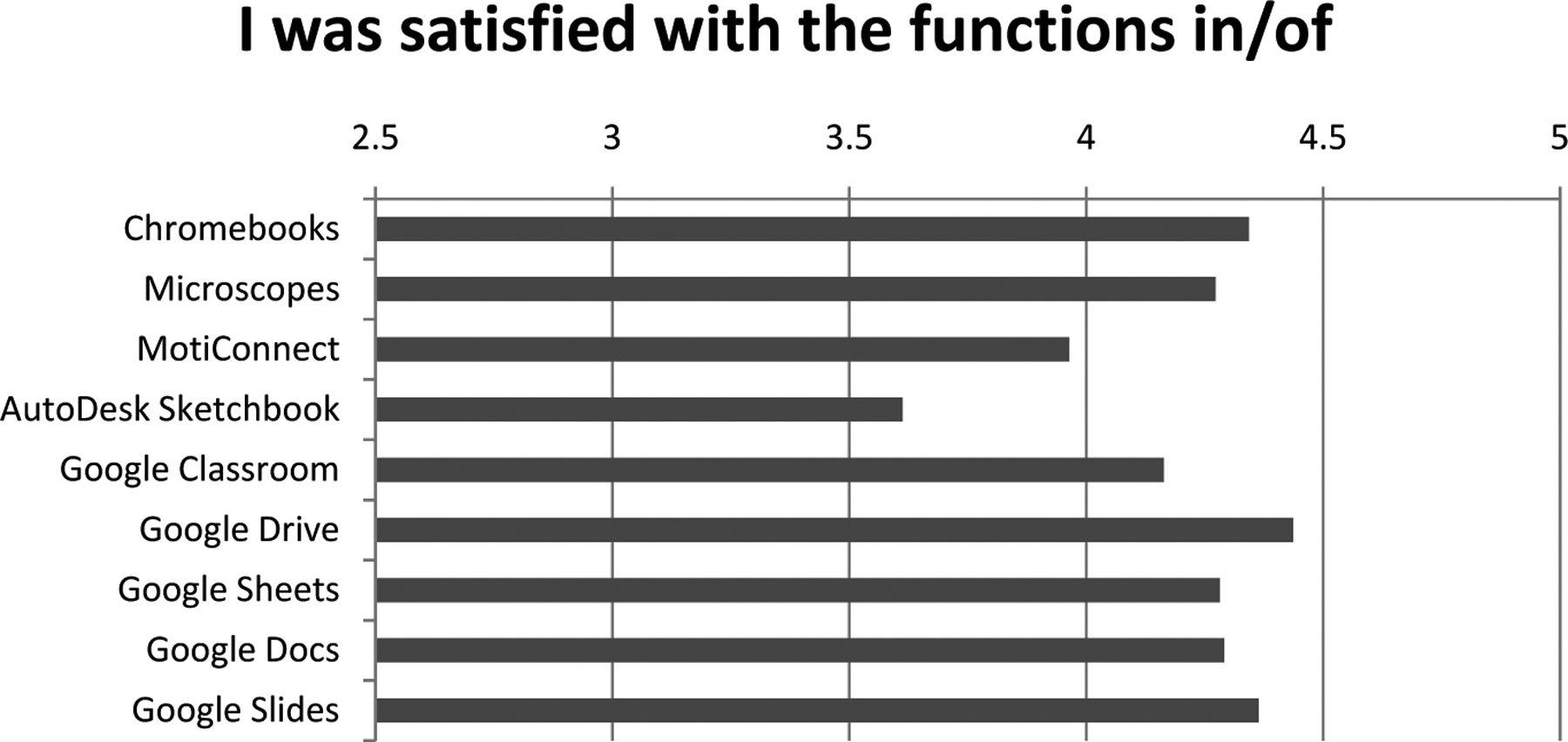
Survey of technology satisfaction results. Survey questions were asked on a 5-point Likert-type scale, with 5 being high and 1 being low (n = 55 responses).

A technology survey to gauge teacher satisfaction with the different technological aspects of our program was also administered (Figure 2). 55 teachers that attended the course completed this assessment, and were asked to indicate their satisfaction with various aspects of each technological tool we used during the course on a 5-point Likert-type scale ranging from “Strongly Disagree” (1) to “Strongly Agree” (5).

Of the technological tools utilized (Chromebooks, Microscopes, MotiConnect, Autodesk Sketchbook, Google Classroom, Google Drive, Google Sheets, Google Docs, and Google Slides), all received mean ratings higher than 3.5, and all but 2 were rated higher than 4. With 3 deemed a “neutral” response, these ratings are consistent with the view reflected from the course evaluation that teachers had an overall positive view of how the course was delivered, including the technological tools utilized. The ratings for Autodesk SketchBook were the lowest of any tool, with a mean rating of 3.61, driving further review of that tool.

As part of this survey, teachers were also given the opportunity to offer narrative feedback on the technological tools used during the course. This feedback was overwhelmingly positive, with many comments positively referencing the Chromebooks, general technology integration, and the support available when using the technological tools. For example, one teacher said “Chromebooks and Google Classroom were AMAZING! I plan on using these things in my classroom for sure next year” and another “it was great to incorporate technology!” Still other teachers were appreciative of the opportunity to learn how to use new technological tools, saying they “provided a new learning opportunity” and “I appreciate the ability to use tools that I would not normally have the opportunity to use”. In addition to valuing the technological tools, one teacher referenced their value within general teaching practice, saying “It was another experience where teachers might struggle and can relate to how their students also struggle through learning experiences”.

### 3.3. Technology Use Self-Efficacy

Teachers were also asked to reflect on their comfort using technology in the classroom before and after the course on a 5-point Likert-like scale (Table 3). Mean rating for teachers was 3.95 before the course and 4.31 after the course. This difference is statistically significant, t(54) = 3.83, *p* < 0.001, which suggests that teachers’ perceived self-efficacy in using technology for work increased as a result of the course.

## 4. Discussion

### 4.1. Overall

Overall, this conversion to a digital delivery of a 12-day course curriculum was well received by the teachers. The positive course and technology satisfaction surveys substantiate this statement, and support an overarching valuation of the content and digital delivery of the curriculum by the teachers who participated.

### 4.2. Teacher Self-Efficacy

There were several major benefits to digitizing the curriculum that impacted both the teacher learners and facilitators. One of the most interesting and, perhaps surprising, benefits was the increase in teachers’ perceived self-efficacy in regards to using technology at work, which was corroborated by teacher statements about appreciation of use of new tools and intention to use those tools in the classroom in the future. This is of key importance as supporting student learning with technology is best when teacher-driven (Miranda & Russell, 2012), and teachers often lack confidence in utilizing technology in the classroom (Moore-Hayes, 2008, 2011).

### 4.3. Course Revision

In addition to this growth in comfort with technology, there are other improvements to the course itself. The digital format of the curriculum allows the schedule and content to be adjusted and revised as needed to optimize learning. The digital interaction with the scientific process is also an improvement, as it allows for instantaneous transfer of experimental data via Chromebooks and the Google Drive. It also provides for more effective collaboration on experimental writing work using Google Docs. In addition, the synchronization of course delivery with experimental data collection and presentation makes it easier for teachers involved in the process to apply lessons from the classroom and codeswitch between learning and application.

When assessing course effectiveness, collecting surveys digitally provides instantaneous feedback and streamlined data analysis. This ultimately makes the data much more useful for altering delivery, as the digital platform reduces the labor-intensive analysis typically needed following our course runs. Overall, this has enabled immediate feedback and focus on implementing the feedback for subsequent course deliveries, even within the same season, which was not feasible prior to digitization.

### 4.4. Continued Challenges

Not surprisingly, there are areas that will need improvement. The organization of the Google Classroom has been revised and improved for future implementations, as a better understanding of how the teachers access the information is developed. Additionally, the initial application utilized for the talking drawing (Clarisketch) caused the most direct concern amongst the teachers partly due to the drawing capability as well as the steps needed to complete the assessment. AutoDesk Sketchbook was an improvement, but remains the lowest rated technological tool. The importance of the talking drawings to InSciEd Out’s goals to promote creativity and language production places this digital tool as a high priority for improvement. It is a concern if there is discomfort using technology for the assessment, as it will likely hinder the goals of the assessment.

## 5. Conclusion

The integration of technology will continue to benefit InSciEd Out in future professional development programming. While there was a significant time and cost investment up front, this has easily been outweighed by the long-term benefits of increased flexibility and use of the technology for future iterations of the course. Teachers had a high level of satisfaction with the course and technology itself. Despite some minor difficulties, particularly with the “Talking Drawing” assessment, converting the professional development to a digital platform was a success for InSciEd Out and may act as a roadmap for other programs transitioning to digital platforms.

## Figures and Tables

**Table 1.** Physical to digital curriculum flowchart.

Content	Pencil & Paper Course	Digital Course
**Curriculum**	Large 3-Ring Binder	Google Classroom & Chromebooks
**Scientific Tools**	Software on single tablet	Software on Individual Chromebooks
**Assessments**	Paper & Pencil	Digital on Chromebooks
**Collaboration**	Small groups, shared computer or individually owned computers	Multiple people collaborate on same Google Doc simultaneously

**Table 2.** Course evaluation questions: mean responses.

Characteristics of the Course:	
Objectives of the course were clear.	3.48
Assigned readings were of appropriate difficulty.	4.54
Assignments were fair.	4.45
I felt safe to share my voice during this course.	4.43
I am glad I took this course.	4.54
Overall, I would rate this course… (scale from 1—Very Bad to 5—Excellent)	4.51

Participants were asked to respond to survey statements on a 5-point Likert-type scale, with 5 being Strongly Agree and 1 being Strongly Disagree (n = 55 responses).

**Table 3.** Perceived efficacy for using technology in the classroom.

I felt comfortable using technology for work:	
before this course	after this course
3.95	4.31*

Participants were asked to respond to survey statements on a 5-point Likert-type scale, with 5 being high and 1 being low (n = 55 responses).

* significantly different from before course score: t(54) = 3.83, *p* < 0.05.
